# Prognostic Value of Immune-Related Multi-IncRNA Signatures Associated With Tumor Microenvironment in Esophageal Cancer

**DOI:** 10.3389/fgene.2021.722601

**Published:** 2021-09-30

**Authors:** Jingjing Pang, He Pan, Chunxiu Yang, Pei Meng, Wen Xie, Jiahao Li, Yueying Li, Shu-Yuan Xiao

**Affiliations:** ^1^ Department of Pathology, Wuhan University Center for Pathology and Molecular Diagnostics, Zhongnan Hospital of Wuhan University, Wuhan, China; ^2^ Department of Pathology, University of Chicago Medicine, Chicago, IL, United States

**Keywords:** esophageal cancer, tumor microenvironment, long noncoding RNAs, risk score, prognosis, tumor mutation burden, drug susceptibility

## Abstract

Esophageal cancer is the eighth most common cancer and the sixth leading cause of cancer death worldwide. Hence, for a better understanding of tumor microenvironment and to seek for novel molecular targets for esophageal cancer, we performed related studies on two histopathological subtypes of esophageal cancer: esophageal squamous cell carcinoma (ESCC) and esophageal adenocarcinoma (EAC). Bioinformatic analyses were conducted based on the RNA-seq, genomic mutation, and clinical data from TCGA and GEO cohorts. We clustered patients into high-immunity and low-immunity groups through the ssGSEA results. The ESTIMATE algorithm was used to evaluate the tumor microenvironment. Patients with high immunity in both ESCC and EAC had lower tumor purity and poor survival. Subsequently, CIBERSORT was performed to learn about the detailed difference of tumor-infiltrating lymphocytes (TILs) between high- and low-immunity patients. Specific increase of M2 macrophages and decrease of activated dendric cells can be observed in ESCC and EAC, respectively. The most enriched functions and pathways of high-immunity patients were immunoglobulin complex, MHC class II protein complex, and allograft rejection according to the GO terms and KEGG. Two prognostic immune-related multi-lncRNA risk models were constructed and validated by ROC curve and PCA in ESCC and EAC. High-risk patients in both subtypes had poor survival, advanced clinical characteristics, and higher drug susceptibility except cisplatin and sorafenib. In addition, the tumor mutation burden (TMB) was positively correlated with the risk level in the ESCC and EAC and showed distinct differences between the two subtypes. In summary, we comprehensively analyzed the tumor microenvironment for two subtypes of esophageal cancer, identified two multi-lncRNA signatures predictive for the prognosis, and explored the possibility of the signatures to forecast drug susceptibility as well as TMB for the first time. The findings may serve as a conceptual basis for innovative strategy of individualized immunotherapy for esophageal cancer.

## Introduction

Esophageal cancer is a highly invasive malignancy with poor prognosis. According to World Health Statistics in 2018, the incidence of esophageal cancer ranked eighth and mortality ranked sixth (Gong et al., 2019). The 5-years overall survival rate is approximately 15% (Talukdar et al., 2018). Poor outcomes in patients with esophageal cancer are related to diagnosis at advanced stages and the propensity for metastases (Thrift, 2021). Esophageal cancer is generally classified into two histopathological subtypes: esophageal squamous cell carcinoma (ESCC) and esophageal adenocarcinoma (EAC). ESCC usually originates from esophageal squamous epithelial cells, always driven by the exposure of tobacco, alcohol, hot drink and malnutrition. In contrast, EAC develops from columnar metaplasia of the lower esophagus and is related to obesity and gastric acid reflux (Fatehi Hassanabad et al., 2020). These two subtypes have striking differences in geographical distribution possibly on account of the differences in exposure to risk factor and lifestyle. ESCC comprises the vast majority of esophageal cancer in southeastern and central Asia, southeastern Africa, and south America, whereas EAC is the predominant subtype in Northern Europe, Western Europe, North America, and Oceania, constituting approximately 46% of the global EAC with more pronounced differences in gender than ESCC (Dong et al., 2018).

Currently, the major therapeutic approaches of esophageal cancer are surgical resection and neoadjuvant chemoradiotherapy (CRT). The most commonly used biological and targeted agents in esophageal cancer included angiogenesis inhibitor ramucirumab and the inhibitors of epidermal-growth-factor receptors trastuzumab (Fatehi Hassanabad et al., 2020). However, due to the heterogeneity of esophageal cancer leading to inherent resistance to chemotherapy, and limited clinical benefits of intervention or targeted therapy, the survival and prognosis of advanced patients remain disappointing. Nowadays, immunotherapy has become a promising treatment approach, which aims to activate the immune system and rely on its intrinsic immune function to kill tumor cells. The immunotherapy includes chimeric antigen receptor T cells (CAR-T) therapy, immune-checkpoint blockade (ICB), oncolytic virus, and tumor vaccines (Baba et al., 2020). ICB has shown strong anti-tumor activity in solid tumors such as malignant melanoma, non-small cell lung cancer, renal clear cell carcinoma, and prostate cancer (Riley et al., 2019). Anti-PD-1/PD-L1 antibodies has brought a historic revolution for immunotherapy. Pembrolizumab has been approved by the U.S. Food and Drug Administration (FDA) to treat PD-L1 positive patients who have progressive disease after second-line therapies. Unfortunately, this anti-PD-1 antibody failed to improve the treatment efficacy in patients with advanced PD-L1-positive esophageal cancer (Shitara et al., 2018). With deeper research on the immunotherapy of esophageal cancer, more and more evidence showed that the complicated tumor microenvironment of esophageal cancer contributed to the intervention of anti-tumor immunoregulation or immunotherapy of esophageal cancer. Nevertheless, the exact mechanisms were not yet elucidated. Thus, a better understanding of antitumor immunity and tumor microenvironment is of utmost importance to improve the efficiency of immunotherapy.

Tumor microenvironment refers to the cellular environment in which the tumor develops, comprising tumor cells, endothelial cells, fibroblasts, immune cells, cytokines, growth factors, and extracellular matrix (Wu and Dai, 2017). Tumor cells can functionally secrete various cytokines, chemokines, and other factors to sculpt the microenvironment resulting in the alteration of the surrounding cells to intricately influence the occurrence and development of tumor. Tumor-associated macrophages, cytokines, IL-1, and complement have emerged as promoters in tumorigenesis, while myeloid cells and innate lymphoid cells are recognized as the potential tumor suppressor. Growing evidence suggested that the tumor microenvironment plays a pivotal role in regulating immune responses, facilitating immune escape, promoting angiogenesis, and inducing metastasis, contributing to a far-reaching impact on the effectiveness of immunotherapy (Meurette and Mehlen, 2018; Hinshaw and Shevde, 2019; Jarosz-Biej et al., 2019). Meanwhile, tumor mutation burden (TMB) was discovered as a novel biomarker to predict the efficacy of ICB. Higher TMB was generally related to better overall survival after ICB therapy for a variety of cancers, including non-small cell lung cancer, colorectal cancer, bladder cancer, and melanoma (Bader et al., 2020). Concerning the conventional reliable markers in immunotherapy of esophageal cancer, PD-L1 immunohistochemical evaluation results revealed that it hardly accurately predicted the therapeutic response to anti-PD-1 antibody in esophageal cancer patients (Yang et al., 2020a). Based on it, TMB is emerging as an immune-response biomarker for esophageal cancer.

Recently, researches have indicated that lncRNA may also be involved in tumor microenvironment remodeling in esophageal cancer, suggesting that it is of great value to study lncRNA associated with immunity (Robinson et al., 2020). Long non-coding RNAs (lncRNAs) are single-stranded RNAs longer than 200 nucleotides without protein coding potential, which mainly functions as the regulators of chromatin dynamics and gene regulation, closely associated with transcription, translation, and epigenetic modification (Qian et al., 2019). Aberrant expression, mutations, and SNPs of lncRNA are supposed to be correlated to tumorigenesis and metastasis (Wang et al., 2021). Genome-wide association studies have identified a large number of lncRNAs that may serve as biomarkers and therapeutic targets for esophageal cancer.

In this study, we aimed to plot the comprehensive landscape of tumor microenvironment and explore the prognostic immune-related multi-lncRNA signatures in ESCC and EAC. We hope that our findings will help elucidate the pathological mechanism partly and make further contribution to esophageal cancer.

## Materials and Methods

### Data Sources

The publicly available esophageal cancer patient datasets were directly downloaded from The Cancer Genome Atlas (TCGA) data portal (https://tcga-data.nci.nih.gov/tcga/dataAccessMatrix.htm) and Gene Expression Omnibus (GEO, https://www.ncbi.nlm.nih.gov/geo/), which contained RNASeqV2 normalized gene expression data of 135 ESCC samples (TCGA: 95, GEO: 53) and 143 EAC samples (TCGA: 87, GEO: 56) in total. The datasets of GEO originated from GSE54994 and GSE20154. The tumor somatic mutation data of two subtypes of esophageal cancer were also obtained from TCGA and GEO. Clinical information for 185 TCGA esophageal cancer cohorts was downloaded from UCSC Xena (http://xena.ucsc.deu/).

### Single Sample Gene Set Enrichment Analysis

We calculated the enrichment levels of 29 immune-associated datasets in each esophageal cancer sample in the form of ssGSEA scores. These 29 immune signatures containing diverse immune cell types, function, and pathways were obtained from previous publications (Kobayashi et al., 2020) (Supplementary Table S1). Unsupervised hierarchical clustering was performed to classify patients into two subtypes: high immunity and low immunity.“GSVA” R package was used to do the cluster analysis.

### Analysis for Tumor Environment and Immune Infiltration

Estimation of STromal and Immune cells in MAlignant Tumor tissues using Expression data (ESTIMATE) is a tool for predicting tumor purity and presence of infiltrating stromal/immune cells in tumor samples. ESTIMATE algorithm was executed on the basis of ssGSEA results and generated three scores: stromal score (that captures the presence of stroma in tumor tissue), immune score (that represents the infiltration of immune cells in tumor tissue), and estimate score (that infers tumor purity) by “ESTIMATE” R package (Kang et al., 2020).

CIBERSORT algorithm is an R/Web-based tool for deconvolving the expression matrix of human immune cells based on linear support vector regression. Gene expression profiles of 22 common immune cells were downloaded as reference marker from CIBERSORT (https://cibersortx.stanford.edu/). The abundances of the 22 immune cells in esophageal cancer patients were calculated with “CIBERSORT” R package (Yang et al., 2021).

The immune infiltration analysis between high- and low-risk patients was conducted based on the file named “infiltration_estimation_for_tcga.csv” downloaded from TCGA including the immune infiltration data calculated by TIMER, CIBERSORT, CIBERSORT-ABS, EPIC, quanTIseq, MCP-counter, and xCell.

### Building Risk Prediction Model for Esophageal Squamous Cell Carcinoma and Esophageal Adenocarcinoma

Univariate Cox proportional risk regression analysis was performed for each immune-related lncRNA with survival data. Least Absolute Shrinkage and Selection Operator (LASSO)-penalized Cox regression was utilized one step forward to obtain the best candidates of multi-lncRNA for predicting prognosis through the use of the “glmnet” package in R software. Afterwards, a risk score model of the prognostic multi-lncRNA was established according to the following formula: Lasso Risk core = $\overset{n}{\underset{i=1}{\sum }}Coef_{i}*Exp_{i}$ .

### Verification Study

In the validation of the risk model, receiver operating characteristic (ROC) curve and Principal Components Analysis (PCA) were conducted. We used the R package “survival ROC” for time-dependent ROC curve analysis. PCA served as a dimensionality reduction algorithm, utilizing the matrix of normalized gene counts of lncRNA in the risk model. Through orthogonal transformation, we maximized accuracy and minimized the error of overfitting, establishing the correlation between patients with high and low risk. The outcome was visualized using “scatterplot3d” package in R software.

### Evaluation of Drug Sensitivity

IC_50_ represented the concentration necessary for 50% inhibition. We calculated IC_50_ of drugs through “pRRophetic” R package and its dependencies including “car, ridge preprocessCore, genefilter and sva”, which contained the effect information of 138 drugs. The boxplot was plotted by the use of “ggplot2” R package (Song et al., 2020).

### Calculation of Tumor Mutation Burden Scores

Estimation was practiced to count the average number of somatic mutations in tumor genome including coding base substitutions insertions or indels per megabase (Mb) of the sequence examined based on the annotated list from TCGA-ESCA and GEO. We took 38 Mb as a routine value of the length of the human exon and divided the total mutation counts of missense, nonstop, nonsense, and frameshift number by 38 to compute TMB scores. “maftools” R package was employed to draw the waterfall plotting illustrating the relationship between risk scores and TMB in esophageal cancer patients (Kang et al., 2020).

### Statistical Analysis

Statistical analysis was performed with R software version 4.0.4 on R Studio and GraphPad Prism eight software. The RNASeqV2 normalized gene expression and somatic mutation data from TCGA and GEO was merged by the “limma” R package and batch effect was removed by the “sva” R package. The differentially expressed immune-related lncRNAs were screened out with the application of the “limma” R package. Cox regression and survival analysis were conducted through “survival” and “survminer” R package. Hazard ratios for univariate and multivariate analysis were calculated by Cox proportional hazards regression model. The “pheatmap” R package was used for plotting heatmaps in analyses of cluster and risk score model. Wilcoxon rank-sum test was used to compare the difference of two groups of quantitative data; *p* < 0.05 was considered significant. Fisher’s exact test was used to analyze the ratio difference of immunity and risk cluster. The Gene ontology (GO) terms and KEGG pathways were analyzed by the R package “clusterProfiler”, which were identified by a threshold of *p* < 0.05.

## Results

### Tumor Immune Microenvironment in Esophageal Squamous Cell Carcinoma and Esophageal Adenocarcinoma

**FIGURE 1:**
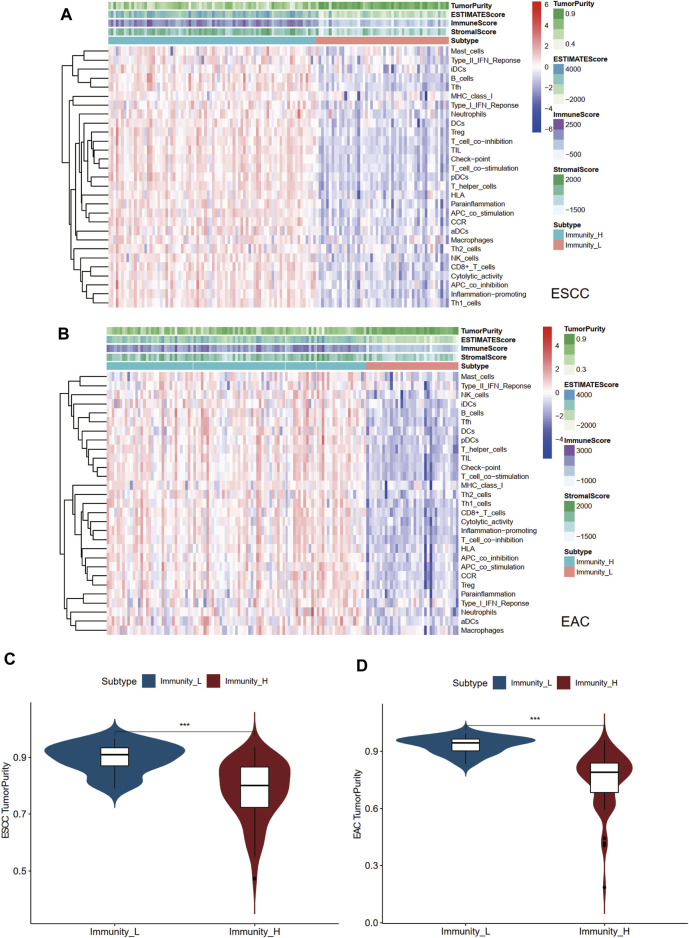
Landscape of tumor immune microenvironment in ESCC and EAC. **(A)** Heatmap of the tumor purity, ESTIMATE scores, stromal scores, immune scores, and tumor-infiltrating lymphocytes in Immunity_L and Immunity_H groups in ESCC. **(B)** Heatmap of the tumor purity, ESTIMATE scores, stromal scores, immune scores, and tumor-infiltrating lymphocytes in Immunity_L and Immunity_H groups in EAC. **(C)** Difference of tumor purity between Immunity_L and Immunity_H groups in ESCC. **(D)** Difference of tumor purity between Immunity_L and Immunity_H groups in EAC. ESCC, esophageal squamous cell carcinoma; EAC, esophageal adenocarcinoma; ssGSEA, single sample Gene Set Enrichment Analysis; ESTIMATE, Estimation of STromal and Immune cells in MAlignant Tumor tissues using Expression. *p < 0.05; **p < 0.01; ***p < 0.001.

Two immune clusters of esophageal cancer, namely, Immunity high (Immunity_H) and Immunity low (Immunity_L), were grouped according to the enrichment scores of infiltrating immune cells, immunity functions, and pathways between ESCC and EAC samples by the ssGSEA (Supplementary Table S2). Then, following the ESTIMATE algorithm, tumor purity, immune score, stromal score, and ESTIMATE score were calculated to further explore the tumor microenvironment. As a result, the stromal, immune, and ESTIMATE scores of the Immunity_H group were markedly higher than those in the Immunity_L group in both ESCC and EAC (Figures 1A,B). Conversely, the Immunity_L group scored higher in tumor purity (Figures 1C,D). The heatmap demonstrated the differential stromal/immune cell infiltration between the two clusters respectively in ESCC and EAC (Supplementary Table S3).

### Expression Level of Human Leukocyte Antigens Family Genes and Immune Checkpoints

**FIGURE 2:**
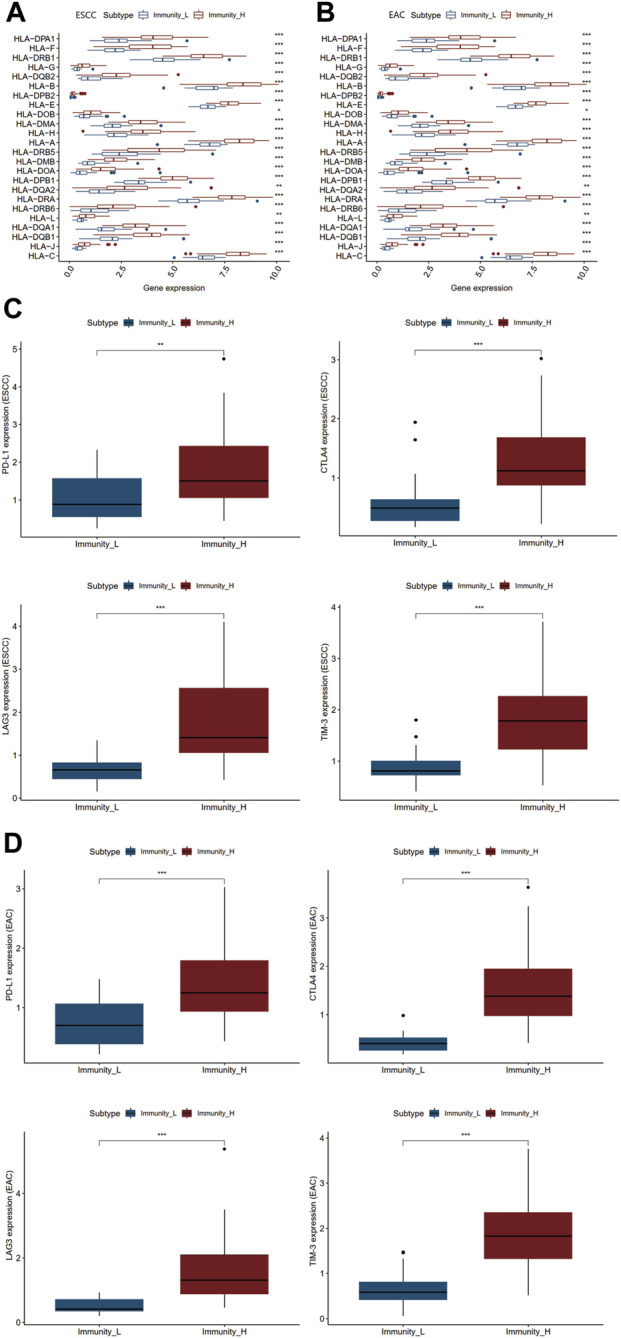
Differential genetic analyses of two immunity clusters in ESCC and EAC. **(A)** Differential expression of HLA family genes in Immunity_L and Immunity_H groups of ESCC. **(B)** Differential expression of HLA family genes in Immunity_L and Immunity_H groups of EAC. **(C)** Differential expression of PD-L1, CTLA4, LAG3, and TIM-3 in Immunity_L and Immunity_H groups of ESCC. **(D)** Differential expression of PD-L1, CTLA4, LAG3, and TIM-3 in Immunity_L and Immunity_H groups of EAC. *p < 0.05; **p < 0.01; ***p < 0.001.

Human leukocyte antigens (HLAs) mostly encoded by human major histocompatibility complex (MHC) were deeply involved in the resistance to foreign pathogens and immunological responses in a variety of pathological processes. Immune checkpoints have been proved to be crucial therapeutic targets for ICB (Wu et al., 2020a). We inspected the differential HLA genes and identified four immune checkpoint genes PD-L1, CTLA4, LAG3, and TIM-3 strongly associated with ESCC and EAC (Huang and Fu, 2019). As shown in Figures 2A–D, the expression levels of both HLA-related genes and immune checkpoints were notably elevated in the Immunity_H group in ESCC and EAC (Supplementary Table S4).

### Prognostic Features of the Two Immune Clusters in Esophageal Squamous Cell Carcinoma and Esophageal Adenocarcinoma

**FIGURE 3:**
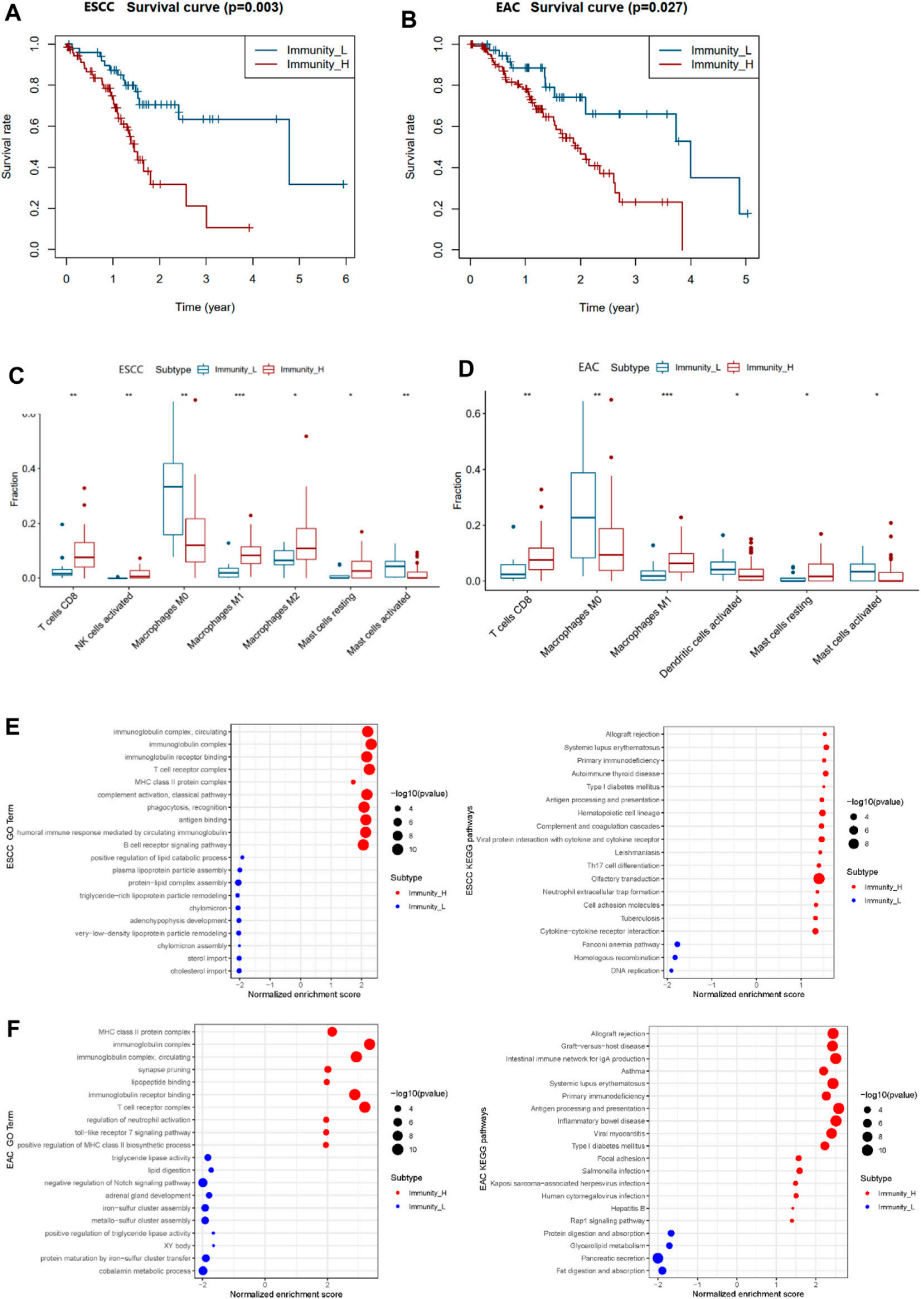
Differential survival, TILs, and enriched functions and pathways of two immunity clusters in ESCC and EAC. **(A)** Survival curve of Immunity_L and Immunity_H groups in ESCC. **(B)** Survival curve of Immunity_L and Immunity_H groups in EAC. **(C)** Boxplot of differential TILs in Immunity_H and Immunity_L in ESCC. **(D)** Boxplot of differential TILs in Immunity_H and Immunity_L in EAC. **(E)** The most enriched GO terms and KEGG pathways between Immunity_L and Immunity_H groups in ESCC. **(F)** The most enriched GO terms and KEGG pathways between Immunity_L and Immunity_H groups in EAC. TILs, tumor-infiltrating lymphocytes; GO, Gene ontology; KEGG, Kyoto Encyclopedia of Genes and Genomes. *p < 0.05; **p < 0.01; ***p < 0.001.

The survival data of patients with ESCC and EAC were used for overall survival analysis. The survival curve was plotted in Figures 3A,B showing that Immunity_L patients had longer survival than Immunity_H patients. The same situations occurred in ESCC and EAC (Supplementary Table S5).

### Tumor-Infiltrating Lymphocytes in the Two Immune Clusters in Esophageal Squamous Cell Carcinoma and Esophageal Adenocarcinoma

In order to investigate the differences of the tumor-infiltrating lymphocytes (TILs) between the two immune clusters, CIBERSORT was used to calculate the fractions of 22 TILs, respectively (Supplementary Table S6). Finally, we identified seven TILs of significant differences in ESCC and six TILs in EAC (Figures 3C,D). In ESCC, CD8+T cells, activated NK cells, M1 macrophages, M2 macrophages, and resting mast cells appeared more in the Immunity_H group than in the Immunity_L group, while the M0 macrophages and activated mast cells markedly decreased. In the Immunity_H group of EAC, CD8+T cells, M1 macrophages, and resting mast cells increased with the amounts of M0 macrophages, activated dendritic cells, and activated mast cells declining.

### GO Terms and KEGG Pathways of the Two Immune Clusters in Esophageal Squamous Cell Carcinoma and Esophageal Adenocarcinoma

To further evaluate the similarities and differences of immunological functions between the two immune clusters of esophageal cancer, GO terms and KEGG pathway enrichment analysis was carried out. As displayed in Figures 3E,F, the main functions of the Immunity_H group enriched were similar in ESCC and EAC patients, including the “MHC class II protein complex”, “immunoglobulin complex”, “immunoglobulin receptor binding”, and “T cell receptor complex”. The major enriched KEGG pathways in the Immunity_H group of ESCC and EAC were associated with immune response. The most significant pathway in ESCC and EAC was “Allograft rejection”. Moreover, there were some cancer-related pathways among the ESCC and EAC KEGG list, such as “cytokine-cytokine receptor interaction”, “DNA replication”, “focal adhesion”, and “Rap1 signaling pathway” (Supplementary Table S7).

### Construction of Multi-lncRNA Risk Assessment Model for Esophageal Squamous Cell Carcinoma and Esophageal Adenocarcinoma

Based on the RNA-sequencing data of TCGA and GEO samples from TCGA and GEO, we removed the genes encoding proteins and collected lncRNA expression data separately. Then, we choose the significantly differential and highly correlated with immune genes filtered by the criteria of Log|FC| > 1 and correlation coefficient R ≥ 0.4. *p* < 0.05 was set as the significance threshold. Subsequently, we incorporated the survival data into the immune-related lncRNA expression matrix and applied univariate Cox regression analysis to characterize lncRNA with good predictive performance for prognosis. As a result, differentially expressed immune-related lncRNA associated with prognosis were screened out with *p* < 0.05 in both ESCC and EAC (Supplementary Table S8).

**FIGURE 4:**
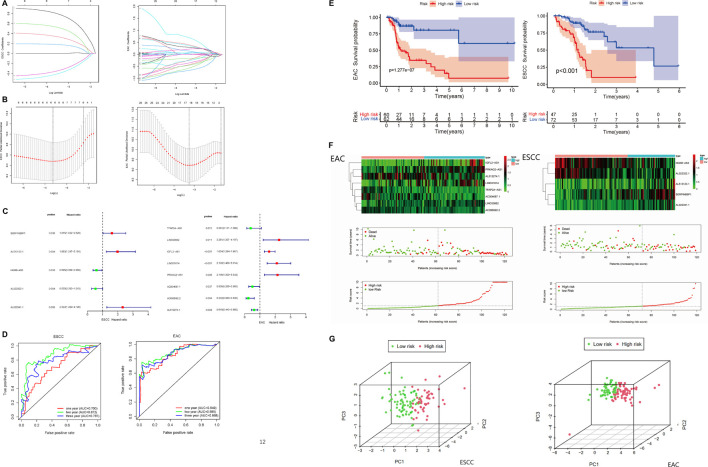
Construction of the prognostic immune-related multi-lncRNA risk model in ESCC and EAC. **(A)** Lasso regression coefficient profiles of the prognostic lncRNA in ESCC and EAC. **(B)** Optimal and minimum criterion of Lasso penalization parameter (λ) used 10-fold cross-validation in ESCC and EAC cohorts. **(C)** Multi-lncRNA prognostic signatures in ESCC and EAC. **(D)** ROC curve of 1-, 2-, and 3-years overall survival for validating the capacity of predicting prognosis of the risk models. **(E)** Kaplan–Meier survival analyses in “High-risk” and “Low-risk” groups of ESCC and EAC. **(F)** Expression level of the lncRNA and patients’ survival status in risk models of ESCC and EAC. **(G)** Principal component analysis (PCA) in patients of “High-risk” and “Low-risk” groups based on the immune-related lncRNA of risk models in ESCC and EAC. ROC, receiver operating characteristic. AUC, area under curve.

To further assess the prognostic value of immune-related lncRNA, the Lasso Cox regression analysis was conducted. We identified the variation of regression coefficients for the prognostic lncRNA and selected the optimal and minimum criterion of penalization parameter (λ) used 10-fold cross-validation (Figures 4A,B, Supplementary Table S9). According to the Lasso risk score formula, we picked out the most correlated lncRNA with survival to establish the multi-lncRNA survival risk score models (Figure 4C). A 5-lncRNA signature of ESCC and an 8-lncRNA signature of EAC were obtained. SERPINB9P1, AL513123.1, and AL022341.1 were the risk factors of ESCC, while HOXB-AS3 and AL022322.1 were the protective factors. In EAC, LINC00662, IGFL2-AS1, LINC01614, and PRKAG2-AS1 were the risk factors, when TFAP2A-AS1, AC004687.1, AC096992.2, and AL512274.1 were protective factors. ROC analysis of the risk assessment model was performed to estimate the specificity and time-dependent sensitivity for survival risk groups (Figure 4D). The 1-year, 2-years, and 3-years area under curve (AUC) for the multi-lncRNA risk score model in ESCC and EAC suggested that these two models can effectively evaluate the prognosis of esophageal cancer patients. ESCC and EAC patients were grouped into “High risk” and “Low risk” according to the cutoff values from ROC analysis. Kaplan–Meier survival curves were plotted to help visualize the survival performance more intuitively. The results showed that the overall survival of ESCC and EAC patients in the “High-risk” group was significantly poorer than that in the “Low-risk” group (Figure 4E). In Figure 4F, it presented the expression level of lncRNA and patients’ survival status in the risk model. With the elevation of risk scores, the survival time declined and the number of deaths increased.

Furthermore, PCA was used to validate the accuracy of the multi-lncRNA risk models. According to Figure 4G, “High-risk” and “Low-risk” patients were separated completely in the multi-lncRNA risk models, which meant the risk models have good differentiability for patients.

### Clinical Traits of Multi-lncRNA Signatures in Esophageal Squamous Cell Carcinoma and Esophageal Adenocarcinoma

**FIGURE 5:**
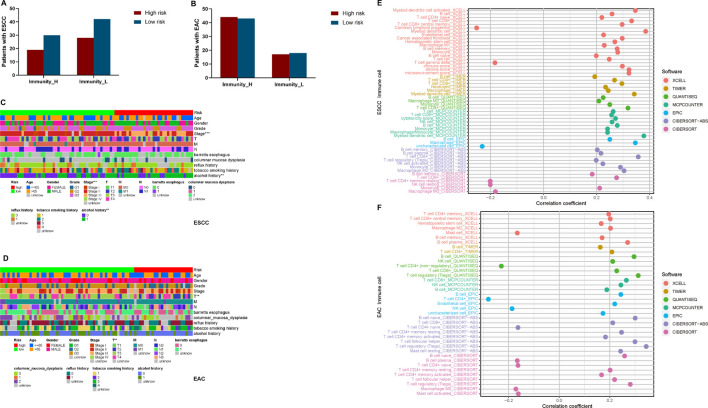
Clinical traits and TILs correlated analyses for esophageal cancer patients. **(A)** The constituent proportion of high- and low-risk patients in immunity clusters in ESCC. **(B)** Constituent proportion of high- and low-risk patients in immunity clusters in EAC. **(C)** Heatmap of clinical traits correlated with risk in ESCC. **(D)** Heatmap of clinical traits correlated with risk in EAC. **(E)** Correlation between risk and TILs in ESCC. **(F)** Correlation between risk and TILs in EAC. T, Depth of tumor invasion; M, Metastasis; N, Lymph Node; *p < 0.05; **p < 0.01; ***p < 0.001.

To further clarify if the constituent ratio of high- and low-risk patients with esophageal cancer was proportioned in Immunity_H and Immunity_L clusters, we plotted the column charts and performed Fisher’s exact test (Figures 5A,B). There was no significant correlation between immunity and risk level in both ESCC and EAC (*p* > 0.05) (Supplementary Table S10). In addition, we also explored the relationships between risk scores and clinical traits. As shown in Figures 5C,D, stage and alcohol were the most correlative clinical traits with the risk score in patients with ESCC, while in patients with EAC, only T (depth of tumor invasion) stages were observed relative to the risk score.

### TILs of Multi-lncRNA Signatures in Esophageal Squamous Cell Carcinoma and Esophageal Adenocarcinoma

Depending on variate software analyzing the TILs, we identified the association between the risk scores and TILs (Figures 5E,F). We found that most TILs showed positive correlation with risk in both ESCC and EAC. Only common lymphoid progenitors, γδT cells, resting CD4^+^ memory T cells, resting NK cells, and M0 macrophages presented negative correlation in ESCC, while the mast cells, naïve CD4^+^ T cells, and M0 macrophages were negatively related to risk in EAC (Supplementary Table S11).

### Drug Sensitivity of Multi-IncRNA Signature in Esophageal Squamous Cell Carcinoma and Esophageal Adenocarcinoma

**FIGURE 6:**
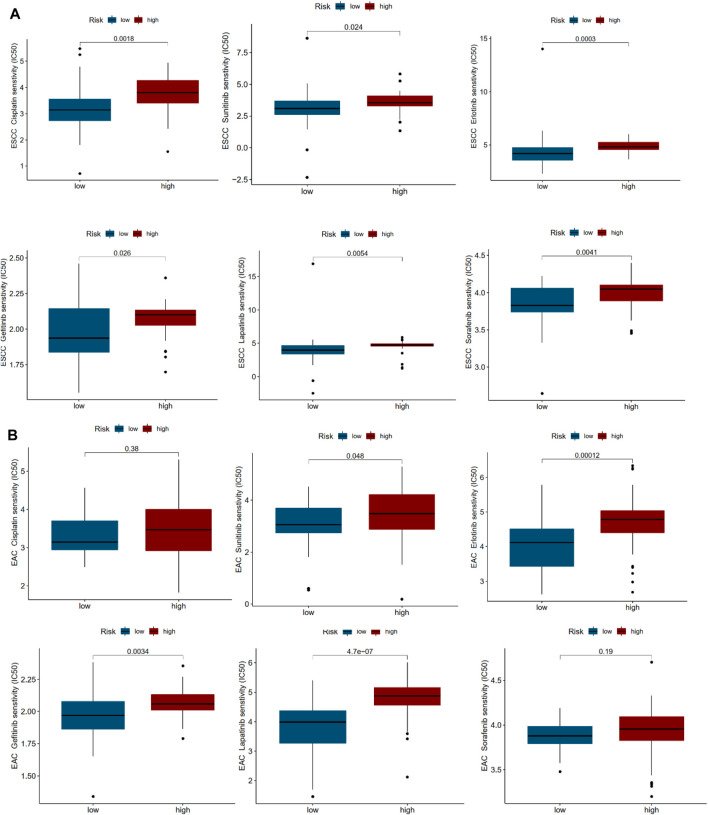
Drug sensitivity correlated with high- and low-risk patients in ESCC and EAC. **(A)** IC_50_ values of various drugs in high- and low-risk patients with ESCC. **(B)** IC_50_ values of various drugs in high- and low-risk patients with EAC.

To investigate the possible application of the multi-lncRNA signature to personalized treatment of esophageal cancer patients, we examined the relationship between risk scores and IC_50_ of drugs universally used or studied in the treatment of ESCC and EAC. These agents included cisplatin, sunitinib, erlotinib, gefitinib, lapatinib, and sorafenib. As shown in Figures 6A,B, high-risk ESCC patients appeared to be more susceptible to most of the drugs than low-risk patients. However, high-risk patients with EAC may not benefit more from cisplatin and sorafenib treatment.

### Tumor Mutational Load of Multi-IncRNA Signatures

**FIGURE 7:**
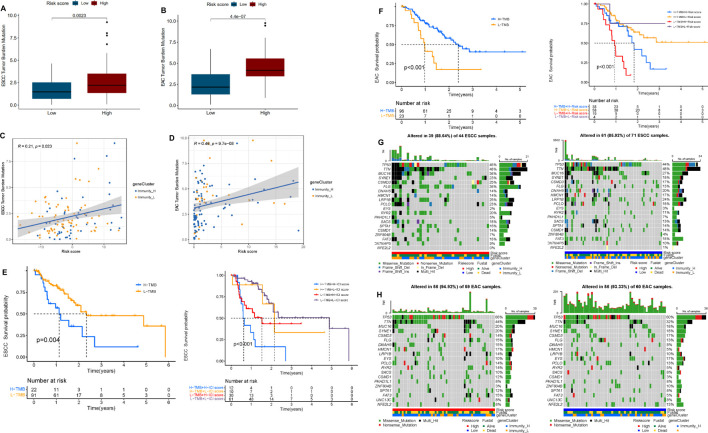
Correlation between tumor mutation burden (TMB) and risk score. **(A)** Differential TMB in high- and low-risk groups in ESCC. **(B)** Differential TMB in high- and low-risk groups in EAC. **(C)** Linear correlation between TMB and risk score in ESCC. **(D)** Linear correlation between TMB and risk score in EAC. **(E)** Survival curves of high and low TMB groups and TMB-risk combined survival curves in ESCC. **(F)** Survival curves of high and low TMB groups and TMB-risk combined survival curves in EAC. **(G)** Waterfall plot of top 20 mutant genes in the high- and low-risk group in ESCC. **(H)** Waterfall plot of the top 20 mutant genes in the high- and low-risk group in EAC.

To understand the potential role of tumor mutational load in esophageal cancer, somatic mutation data of ESCC and EAC patients were collected and corresponding TMB scores were calculated (Supplementary Table S12). The results indicated that in ESCC and EAC patients, high risk always implied high TMB scores. The risk score was positively correlated to the TMB score in EAC (R = 0.46, *p* < 0.05), but weak correlation in ESCC (R = 0.21, *p* < 0.05) (Figures 7A–D). In addition, we identified “High-TMB” and “Low-TMB” groups by a cutoff of the median and performed survival analysis. It revealed that the “TMB-High” group had worse survival than the “TMB-Low” group in ESCC and EAC. Besides, it seemed that there was a combined influence of TMB and multi-lncRNA signature on patient survival outcomes in ESCC and EAC (Figures 6E,F). Waterfall plots showed the concrete mutation differences of the top 20 genes between “High-risk” and “Low-risk” groups in ESCC and EAC (Figures 6G,H). The same with TMB scores, the mutation frequency in high-risk patients was higher. The top 20 genes of ESCC and EAC were distinct in order, and the mutant genes were consistent. TP53 was the gene with the highest mutation frequency in ESCC and EAC. However, in EAC, the mutation of TP53 was more frequent. Compared to EAC, the frameshift deletion or insertion and in-frame deletion were more common in the top 20 mutant genes of ESCC. The mutations in EAC patients were mainly composed of multi-hits and nonsense mutation.

## Discussion

In this study, for a deeper understanding of the tumor microenvironment, we comprehensively analyzed immune infiltration *via* ssGSEA and ESTIMATE algorithms to evaluate the fraction of immune cells in two histopathological subtypes of esophageal cancer ESCC and EAC, respectively. In ESCC and EAC cohorts, the tumor microenvironment was similar to some degree. High-immunity groups always had lower tumor purity and poorer survival. The expression of HLA family members and immune checkpoints was also higher in high-immunity groups. However, there were some differences in TILs, GO terms, and KEGG pathways. In the high-immunity group of ESCC, the amounts of M2 cells would increase characteristically, while in the high-immunity group of EAC, the amounts of activated dendric cells would decline conversely. When the classification criterion turned to risk level. In high risk of ESCC, compared with that of EAC, the amounts of NK cells might decrease. Towards the difference of TILs between ESCC and EAC, it has been widely studied by a lot of researchers. According to the previous studies, the increase of IL-17-releasing mast cells can be a potential prognostic marker and were positively correlated with CD8+T cells and macrophages in the same site in ESCC, indirectly mediating their tumor activity by promoting the recruitment of other effector immune cells (Gong et al., 2019; Han et al., 2020). CD8+T cells in esophageal cancer have been proved to be associated with survival rate, response to neoadjuvant chemotherapy, and lymph node metastasis rate (Zheng et al., 2020). In addition, helper T cells of type 17 (Th17) show contradictory functions in the regulation of esophageal cancer tumor growth. Th17 can promote the invasion of EAC cells but plays a protective role in ESCC by enhancing the cytotoxic effect of natural killer (NK) cells and activating CD1A + DC in tumors (Liu et al., 2017; Melo et al., 2020). Furthermore, tumor-associated macrophages could induce angiogenesis and invasion. Tumor-associated fibroblasts can secrete growth factors, alter the extracellular matrix, and promote tumor migration and metastasis (Kashima et al., 2019). The amounts and activity of TILs are considered to be the key factors to determine the effect of ICB and can predict the prognosis of esophageal cancer (Yagi et al., 2019).

In order to seek for more reasonable prognostic predictors in ESCC and EAC, a prognostic 5-lncRNA signature for ESCC and an 8-lncRNA signature for EAC by Cox regression and Lasso regression analysis, both of which exhibited high accuracy and applicability in predicting the risk of death. We also investigated the clinical traits associated with the risk models of ESCC and EAC. Advanced stages and alcohol consumption were likely correlated with high risk of ESCC patients. Alcohol intake has been testified as an independent risk factor for ESCC currently (Ohashi et al., 2015).

In recent years, studies on lncRNA in esophageal cancer have shown promising results. More and more lncRNAs associated with ESCC or EAC were identified and employed to the diagnosis, prognosis, and therapy. LncRNA CASC9 was considered to upregulate LAMC2 expression and promote ESCC metastasis by interacting with CREB-binding proteins to modify histone acetylation (Liang et al., 2018). Cancer-associated fibroblasts (CAFs) could promote lncRNA DNM3OS to regulate DNA damage reaction, leading to significant radio-resistance (Zhang et al., 2019). LncRNA PVT1A has been proved to serve as a therapeutic target for EAC. Combined targeting of PVT1 and YAP1 might benefit patients with EAC as well. Among the 5-lncRNA signature of ESCC, lncRNA HOXB-AS3 has been found to be abnormally expressed in non-small cell lung cancer, colon cancer, and acute myeloid leukemia (Huang et al., 2017; Huang et al., 2019; Jiang et al., 2020). In the study by Bin et al., TFAP2A-AS1 has been proved to act as a miRNA sponge for miR-933 and regulate the expression of Smad2 (Wu et al., 2020b). In the 8-lncRNA signature, LINC00662 was certified to be upregulated in EAC. It has been proved to accelerate M2 macrophage polarization and hepatocellular carcinoma progression *via* activating Wnt/β-catenin signaling. Its overexpression promoted the occurrence and development of colon cancer by competitively binding with miR-340-5p to regulate CLDN8/IL22 co-expression and activating ERK signaling pathway (Zhou et al., 2019). What is more, LINC00662 is also closely related to gastric cancer, glioma, chordoma, and so on (Liu et al., 2018a; Wu et al., 2020b; Wang et al., 2020). The remaining lncRNAs in the 8-lncRNA signature were associated with lung adenocarcinoma, colon cancer, and so on (Liu et al., 2018b; Cai et al., 2021). The research directed at the lncRNA in the multi-lncRNA signatures of ESCC and EAC was still deficient. Therefore, more studies should be conducted to help explore novel and promising targets for the therapy of esophageal cancer.

Cisplatin, RTKs inhibitors (sunitinib), EGFR inhibitors (erlotinib and gefitinib), HER-2 inhibitor (lapatinib), and multikinase inhibitor (sorafenib) were widely used and studied in the treatment of ESCC and EAC (Yang et al., 2020b). In ESCC, the IC_50_ of drugs mentioned above all presented significant difference in high- and low-risk patients. Nevertheless, in EAC, cisplatin and sorafenib showed no difference in IC_50_ between high- and low-risk patients. The two multi-lncRNA signatures of ESCC and EAC might help speculate the effectiveness of therapeutic agents and contribute to personalized treatments.

Finally, we evaluated the relationship between TMB and the risk level. The results manifested that both of our multi-lncRNA signatures were positively correlated with the TMB. Among the top 20 mutant genes, TP53 mutated more frequently in EAC patients. As reported, the mutation of TP53 might be the early events in the development of EAC by participating the process of chronic gastroesophageal reflux disease (Guo et al., 2018; Dang and Chai, 2020). Prior to TMB being explored as a biomarker, the focus was on quantitative testing of PD-L1 to identify patients who could benefit most from ICB treatment. It is currently approved as an adjunct diagnostic for pembrolizumab in NSCLC (Reck et al., 2016; Mok et al., 2019). However, single use of PD-L1 expression level has gradually shown poor prediction in ICB treatment response, so TMB was developed as a complementary biomarker. A therapeutic benefit dependent on TMB but independent of PD-L1 expression level was observed in patients treated with a combination of nivolumab + ipilimumab and the standard of care (SOC) chemotherapy (Reck et al., 2019). This condition was thought to be present in tumors with high TMB and T-cell infiltration and/or activation regulated in a CTLA-4-dependent manner (Hu et al., 2020). Moreover, TMB has been found to be of predictive value in immunotherapy other than ICB, with studies showing that TIL therapy has better therapeutic outcomes in patients with higher TMB (Liu et al., 2019; Samstein et al., 2019). Hence, our study for TMB might suggest that high-risk patients would be more responsive to immune therapy on account of the high TMB.

## Conclusion

In summary, we have comprehensively examined the characteristics of tumor immune microenvironment in esophageal cancer and identified a 5-immune-related lncRNA signature of ESCC and an 8-lncRNA signature of EAC as the prognostic predictor. These two immune-related lncRNA signatures were validated strictly and appeared to be stable. Additional analysis showed that these two multi-lncRNA signatures could be promising biomarkers to predict drug sensitivity as well as benefits from immunotherapy in esophageal cancer based on TMB.

## Data Availability

Publicly available datasets were analyzed in this study. This data can be found here: The Cancer Genome Atlas (TCGA, https://tcga-data.nci.nih.gov/tcga/) Gene Expression Omnibus (GEO, https://www.ncbi.nlm.nih.gov/geo/) UCSC Xena (http://xena.ucsc.deu/).

## Glossary

APC: antigen-presenting cells
AUC: area under curve
CAF: cancer-associated fibroblasts
CAR-T: chimeric antigen receptor T cells
CRT: chemoradiotherapy
CSC: cancer stem cell
DC: dendritic cells
EAC: esophageal adenocarcinoma
EGFR: epidermal growth factor receptor
ESTIMATE: Estimation of STromal and Immune cells in MAlignant Tumor tissues using Expression
FDA: Food and Drug Administration
GO: Gene Oncology
HER-2: human epidermal growth factor receptor-2
HLAs: human leukocyte antigens
ICB: immune-checkpoint blockade
KEGG: Kyoto Encyclopedia of Genes and Genomes
LASSO: Least Absolute Shrinkage and Selection Operator
LncRNA: long non-coding RNA
MDSCs: myeloid derived suppressor cells
MHC: major histocompatibility complex
NK: natural killer
NSCLC: non-small cell lung cancer
PCA: principal components analysis
RESCC: esophageal squamous cell carcinoma
ROC: receiver operating characteristic
RTKs: receptor tyrosine kinases
SOC: standard of care
ssGSEA: single sample Gene Set Enrichment Analysis
TCGA: The Cancer Genome Atlas: The Cancer Genome Atlas
TCGA: The Cancer Genome Atlas: The Cancer Genome Atlas
Th17: helper T cells of type 17
TILs: tumor-infiltrating lymphocytes
TMB: tumor mutation burden
